# Exogenous ALA promotes tomato fruit quality and pigment metabolism: physiological mechanisms

**DOI:** 10.3389/fnut.2025.1729358

**Published:** 2026-01-14

**Authors:** Jiaqi Chen, Ruirui Li, Junwen Wang, Junfang Feng, Yongmei He, Peng Bai, Xingpan Shang, Yue Wu, Jihua Yu, Zhongqi Tang, Guobin Zhang, Jianming Xie, Jing Zhang, Jian Lyu

**Affiliations:** 1 College of Horticulture, Gansu Agricultural University, Lanzhou, China; 2 State Key Laboratory of Aridland Crop Science, Gansu Agricultural University, Lanzhou, China

**Keywords:** 5-aminolevulinic acid, carotenoids, chlorophyll, fruit quality, tomato

## Abstract

Fruit pigmentation serves as a critical phenotypic indicator of commercial quality in tomato. The strategic application of exogenous plant growth regulators has emerged as a sustainable approach for quality enhancement. 5-Aminolevulinic acid (ALA), as a natural plant growth regulator, has been demonstrated to promote plant growth and enhance fruit quality. In this study, the impact of ALA on the color and quality changes during tomato (*Solanum lycopersicum* cv. 184) fruit red ripe was investigated. Fruit at the fruit setting stage was treated with 200 mg·L^−1^ALA, and the content of intermediate products in chlorophyll and carotenoid synthesis, as well as the activities of related enzymes and gene expression levels, were dynamically monitored. Additionally, color parameters of fruit peel, soluble sugar and organic acid components were determined. Results indicated that ALA significantly upregulated the expression of genes related to chlorophyll degradation (including *SlCLH2, SlPPH*, and *SlSGR*). Meanwhile, the expression levels of genes involved in carotenoid synthesis upregulated by exogenous ALA, and the activities of enzymes were also significantly enhanced, including PDS, PSY, and LCYB, which leading to higher levels of lycopene, β-carotene, and other carotenoids accumulated in tomato fruits, thereby improving changes of fruit color. Besides, 200 mg·L^−1^ ALA treatment significantly increased the content of glucose and fructose in the fruit, while reducing the content of malic acid and citric acid, thus enhancing the sugar-acid ratio of the fruit. In conclusion, treatment with 200 mg·L^−1^ ALA can effectively promote the carotenoids biosynthesis and accumulation while improving fruit flavor quality.

## 1 Introduction

During tomato fruits ripening, significant changes occur in their color, nutritional quality, and flavor characteristics (1). With growing market demands for higher quality tomatoes, fruit color has become a key indicator of commercial value. The color change in tomato fruits is influenced by external environmental factors and hormones (2). Previous research has found that treating tomato fruits with appropriate concentrations of abscisic acid and gibberellins promoted the accumulation of lycopene in the fruits (3). Abscisic acid and ethylene have been proposed to play a synergistic role in the color formation of tomato fruits (4). The color transition is mainly attributed to chlorophyll degradation and carotenoid accumulation (5). The synthesis of both chlorophyll and carotenoids involves the participation of geranylgeranyl pyrophosphate (GGPP). On one hand, GGPP is involved in the synthesis of the phytol side chain of chlorophyll through the action of geranylgeranyl pyrophosphate synthase. On the other hand, it serves as a substrate for the biosynthesis of carotenoids (6). It has been reported that the metabolic pathways of carotenoid and chlorophyll biosynthesis in plants influence each other. By inhibiting lycopene cyclase, the precursor substances for chlorophyll synthesis can accumulate significantly in plant tissues (7).

5-Aminolevulinic acid (ALA), as an essential precursor for the synthesis of tetrapyrrole compounds, can enhance plant resistance, improve fruit quality, and promote fruit coloration (8). In recent years, it has regarded as a natural plant growth regulator and become a research hotspot (9–11). In research of kiwifruit, foliar application of ALA at appropriate concentrations increased chlorophyll content in the leaves, enhanced soluble sugar content, and improved the appearance quality of the fruit (12). It has been reported that spraying ALA on tomato fruit during its mature green stage promoted the accumulation of carotenoids, mainly lycopene and β-carotene (13). Exogenous application of ALA applications has been reported to increase vitamin C, soluble solids, and sugars apple fruits (14). Moreover, exogenous ALA treatment has been shown to increase anthocyanin content in peach skin, resulting in enhanced fruit coloration (15). Several studies have also shown that ALA promotes postharvest tomato ripening and improves fruit quality by increasing soluble solids and soluble sugars while reducing titratable acidity (16). These findings indicate that ALA possesses significant potential for promoting fruit quality development and coloration. There is a close relationship between fruit quality and color changes (17).

The previous results of our research team (related to improving tomato fruit quality) did not conduct in-depth studies on the mechanism by which ALA promotes fruit coloration. Therefore, in this study, tomato fruits at the fruit setting stage were treated with ALA solution. Dynamic changes in pigment metabolism and fruit quality during ripening were monitored. The chlorophyll synthesis and degradation, as well as carotenoid biosynthesis were analyzed to elucidate the mechanisms underlying ALA induced coloration of tomato fruit. This research is expected to provide theoretical support for the scientific regulation of pigment formation and the improvement of fruit quality.

## 2 Experimental design and methods

### 2.1 Experimental design

**Figure 1:**
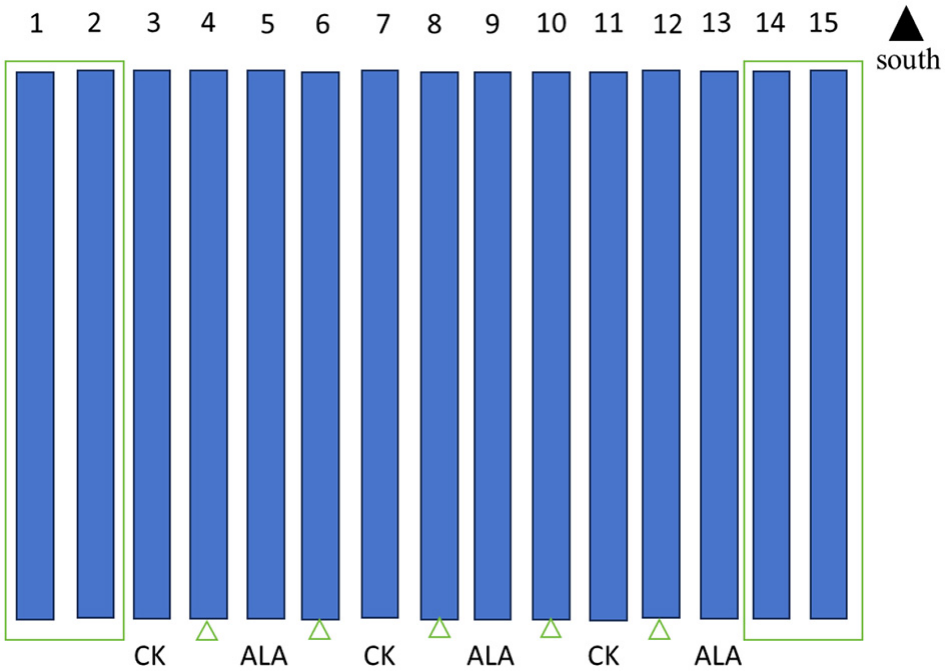
Setting of cultivation tank treatment. Each treatment consisted of 3 plots as biological replicates, and each plot was randomly arranged and the protective plots were set as 1, 2, 4, 6, 8, 10, 12, 14, and 15.

This experiment was conducted in a solar greenhouse at Yuzhong County, Lanzhou City, Gansu Province (35.87°N, 104.09°E). The tomato (*Solanum lycopersicum* cv. 184) seeds were germinated in substrate, and seedlings were transplanted to a solar greenhouse for substrate cultivation when they had developed three fully expanded true leaves. The cultivation substrate was a mixture of peat, vermiculite, and perlite at a volume ratio of 3:1:1. After the flowering of the third inflorescence, the pollination date was recorded. Seven days after pollination, fruits at the fruit setting stage (with a uniform diameter of approximately 1 cm) were selected for exogenous ALA treatment. To ensure stable treatment performance, ALA spraying was conducted at 18:30 after the greenhouse shading curtains were closed, followed by 12 h of darkness. During treatment, the temperature was maintained at 18–28 °C, relative humidity at 50%−65%, and light intensity at 300 mmol s^−1^ m^−2^. Treatments were administered at 10-day intervals until red ripe stage. The experiment included two treatments: (1) spraying distilled water on the fruit surface as a control (CK), and (2) spraying 200 mg·L^−1^ ALA solution on the fruit surface (13). Both treatments included 0.01% Tween-20 as a surfactant. The concentration of ALA used in the treatment was determined based on the optimal concentration obtained from preliminary experiments (13). In the solar greenhouse, each cultivation tank was regarded as one experimental plot. The specifications of the cultivation tank are 9 m in length, 0.4 m in width and 0.25 m in depth. The distance between the troughs is 1 meter. Plant 2 rows of tomato plants in each tank, with 18 plants in each row. The planting density is 36 plants per tank. The spacing between plants in each row is 0.45 m and the row spacing is 0.2 m (Figure 1). For each plot, 15 fruits with consistent growth were marked, and there were a total of 45 fruits in each treatment. Based on the morphological and color changes observed in the fruit development process of the ALA treatment group, the dynamic sampling process was divided into three stages: mature green stage (34–39 days after pollination), breaker stage (45–49 days after pollination), and red ripe stage (55 days after pollination). All treatments were sampled uniformly during these stages.

### 2.2 Measurement indices and methods

#### 2.2.1 Tomato fruit peel color parameters

The color parameters of the tomato fruit peel, including lightness (L^*^), a^*^, b^*^, c, and Hue angle (arctangent, h = b^*^/a^*^), were measured using a CR-10 Plus colorimeter (Konica Minolta Inc., Japan). The L^*^ value of the fruit peel color parameter represents the degree of lightness, ranging from 0 to 100, where 0 indicates black and 100 indicates white, with higher values indicating a brighter color. The a^*^ value indicates the red–green chromaticity. Positive values correspond to increasing redness, with higher values reflecting a more pronounced red hue; negative values correspond to increasing greenness, with lower values indicating a stronger green hue. The b^*^ value represents the yellow-blue chromaticity, ranging from negative to positive, with negative values indicating blue and positive values indicating yellow; the larger the absolute value, the deeper the corresponding color. The c-value represents color saturation, and in the control group, it exhibited a high-low-high trend during the three stages of fruit development. The h value represents the hue angle, with a range from 0° to 180°. An h value of 0°, 90°, and 180° corresponds to purplish-red, yellow, and green, respectively. When h < 50°, a smaller value indicates a deeper red color (18). The color parameter was measured at three points (shoulder, equator, and top) of tomato fruit. Each treatment is repeated three times and the average value is taken.

#### 2.2.2 Indicators related to chlorophyll synthesis and metabolic pathways

The content of endogenous ALA, Protoporphyrin IX (Proto IX), and Mg-protoporphyrin IX (Mg-Proto IX) was measured according to the method of Hodgins and Van Huystee (19), with slight modifications. The tomato fruit sample 5 g was cut into small pieces, and 6 mL of acetate buffer (pH 4.6) was added. The sample was thoroughly ground in an ice bath and centrifuged at 5,000 *g* for 15 min. The supernatant was collected, and 4 drops of ethyl acetoacetate were immediately added. The mixture was condensed at 100 °C for 10 min and then cooled to room temperature. An equal volume of fresh Ehrlich's reagent was added for color development over 15 min. The OD value was measured at 554 nm. The ALA content (nmol·g^−1^ FW) in the sample was calculated using an ALA standard curve.

Fresh tomato fruits were cut into small pieces, and 0.3 g of the sample was weighed. The sample was ground and extracted with 10 mL of 80% alkaline acetone. The mixture was soaked in darkness until the tissue turned white. The supernatant was collected, and the absorbance values were measured at wavelengths of 575 nm, 590 nm, and 628 nm. The contents of Proto IX and Mg-Proto IX (μmol·g^−1^ FW) were calculated using the appropriate formulas.


$$\begin{array}{r} \text{ProtoIX}\left(\text{μ molgF}{\text{W}}^{-1}\right)=0.18016\times \text{A}575-0.04036\times \text{A}628\\ -0.04515\times \text{A}590\times \text{V}/\text{FW}.\\ \text{Mg}-\text{ProtoIX}\left(\text{μ molgF}{\text{W}}^{-1}\right)=0.06077\times \text{A}590-0.01937\times \text{A}575\\ -0.003423\times \text{A}628\times \text{V}/\text{FW}. \end{array}$$

Chlorophyll content was determined following the method of Lichtenthaler et al. (20), with slight modifications. A 2 g sample of fruit tissue was placed in a test tube, and 10 mL of 80% acetone was added. The tube was sealed and left to soak for 48 h until the sample turned white. The supernatant was collected, and absorbance was measured at 646 nm and 663 nm. The chlorophyll content (Chl a and Chl b) was calculated accordingly.


$$\begin{array}{l} \text{Chl a }\left(\text{mg g F}{\text{W}}^{-}1\right)=\left(12.21\times \text{A}663-2.81\times \text{A}646\right)\;\times \text{V}/\text{FW}\\ \text{Chl b }\left(\text{mg g F}{\text{W}}^{-}1\right)=\left(20.13\times \text{A}646-5.03\times \text{A}663\right)\;\times \text{V}/\text{FW} \end{array}$$

#### 2.2.3 Carotenoid components and related enzyme activities

Carotenoid components were determined following the method of Wang et al. (13). A 0.5 g sample of freeze-dried tomato was placed in a brown glass bottle, and 30 mL of a petroleum ether and acetone mixture (2:1, v/v) was added. The sample was extracted under ultrasonic conditions (temperature at 30 °C) for 40 min until all color was removed. The extract was transferred to a separatory funnel and washed twice with 250 mL of ultrapure water. Anhydrous Na_2_SO4 was added to remove the aqueous phase. The extract was evaporated to dryness using a rotary evaporator at 40 °C. The residue was dissolved in a 25 mL mixture of acetonitrile, dichloromethane, and methanol (55:20:25, v/v/v). The solution was filtered through a 0.22 μm organic membrane, and the filtrate was analyzed using High-Performance Liquid Chromatography (HPLC). Compounds were detected at the following wavelengths: 450 nm (β-carotene), 470 nm (phytoene, lycopene), and 286 nm (α-carotene, violaxanthin, and lutein). Quantification was performed based on standard curves. Data analysis was carried out using Empower software (Waters, USA).

The enzyme activities related to carotenoid synthesis in tomato fruits were measured using plant geranylgeranyl pyrophosphate synthase (GGPS, EC 2.5.1.31) quantitative assay kits, plant phytoene synthase (PSY, EC 2.5.1.33) quantitative assay kits, plant phytoene desaturase (PDS, EC 1.14.99.-) quantitative assay kits, plant lycopene β-cyclase (LCYB, EC 5.5.1.19) quantitative assay kits, plant lycopene ε-cyclase (LYCE, EC 5.5.1.18) quantitative assay kits, and plant zeaxanthin epoxidase (ZEP, EC 1.14.13.90) quantitative assay kits (Shanghai Guduo Biotechnology Co., Ltd., China). The specific operations were performed according to the product instructions, with each treatment replicated three times. Enzyme activities were calculated based on the corresponding standard curves.

#### 2.2.4 The sugar components in tomato fruits

Sugar components were extracted following the method of Beckles et al. (21). The tomato fruits to be analyzed were de-stemmed, and an accurate weight of 5 g was taken. The sample was thoroughly ground into a homogenate in a mortar and then transferred to a 50 mL centrifuge tube (rinsing three times) and the volume adjusted to 25 mL. The sample was subjected to ultrasonic shaking at 30 °C for 60 min, followed by centrifugation for 10 min at a speed of 10,000 r·min^−1^ and a temperature of 4 °C. A 1.5 mL aliquot of the supernatant was drawn using a syringe and filtered through a 0.22 μm microporous membrane into a 1.5 mL centrifuge tube for the determination of relevant component contents.

The sugar contents were determined using HPLC, following the chromatographic conditions described by Wilson et al. (22). An Agilent refractive index detector (Agilent series 1100, USA) was used. The chromatographic column was LC-NH2 (460 mm × 250 mm), and the mobile phase consisted of acetonitrile and water in a volume ratio of 3:1. The injection volume was 10 μL, with a flow rate of 1.0 mL·min^−1^ at a column temperature of 30 °C. The contents of fructose, glucose, and sucrose were measured for each treatment, with three replicates per treatment and location.

#### 2.2.5 The organic acid components in tomato fruits

The organic acid content of tomato fruits was determined following the method of Coelho et al. (23), with slight modifications. A 5 g sample of tomato fruit was weighed, thoroughly ground into a homogenate using a mortar, and transferred to a 50 mL centrifuge tube (rinsed three times), then the volume was adjusted to 25 mL. The sample was centrifuged at 10,000 r·min^−1^ for 10 minutes at 4 °C. After centrifugation, use a syringe to extract 1.5 mL of the supernatant and filtered through a 0.22 μm microporous membrane into a 1.5 mL brown injection vial for the determination of relevant component contents.

The organic acid content was determined using HPLC. An Agilent C14 column (300 mm × 7.7 mm) was used, with a UV detector set to a wavelength of 210 nm. The mobile phase was 0.2 mmol·L^−1^ sodium dihydrogen phosphate. The injection volume was 10 μL, with a flow rate of 1.2 mL·min^−1^, and the column temperature was maintained at 30 °C. The contents of malic acid, citric acid, tartaric acid, oxalic acid, and ascorbic acid in tomato fruits were measured.

#### 2.2.6 Total RNA extraction and gene expression levels

Total RNA from tomato fruits was extracted using the RNAprep Pure Plant Plus Kit (TIANGEN Biotech Co., Ltd., China), following the manufacturer's instructions strictly. The cDNA was synthesized using the HiScript II Q RT SuperMix for qPCR (+gDNA wiper) reverse transcription kit (Vazyme Biotech Co., Ltd., China), following the manufacturer's instructions strictly. Then, qRT-PCR was performed using the SYBR Green Pro Taq HS Premixed qPCR Kit (with ROX) (Accurate Biotechnology Co., Ltd., China), and quantitative analysis was conducted using the 2^−ΔΔ*Ct*^ method. *Mg-chelatase subunit CHLH* (*SlCHLH*)*, Mg-protoporphyrin IX methyltransferase (SlCHLM), protochlorophyllide oxidoreductase (SlPOR)*, and *chloroplast signal recognition particle component* (*SlCAO*) are key genes encoding enzymes involved in the chlorophyll synthesis pathway. *Chlorophyllase 2* (*SlCLH2*)*, pheophytinase (SlPPH), pheophorbide a oxidase (SlPAO)*, and *STAY-GREEN (SlSGR)* are key enzyme-encoding genes involved in the chlorophyll degradation pathway. *Geranylgeranyl pyrophosphate synthase* (*SlGGPPS*)*, phytoene synthase 1*(*SlPSY1*)*, phytoene synthase 2* (*SlPSY2*)*, phytoene desaturase* (*SlPDS*), and *lycopene beta-cyclase* (*SlLCYB*) are key enzyme-encoding genes involved in the carotenoid biosynthesis pathway. The tomato *SlActin* gene was used as the internal reference gene, and the primer sequences are shown in Table 1.

**Table 1:** Primer sequences for qRT-PCR.

**Gene**	**Accession number**	**Forward primer 5'−3'**	**Reverse primer 5'−3'**
*SlCHLH*	XM_004236562.4	TTCTCAAACGCTTCGGGTTCTTAC	AGCACCAGGAGCATCACAGTC
*SlCHLM*	XM_004235797.4	CTCCGCCGCTACCGACATC	GACCTCCTCCGCCTGAAGTTG
*SlPOR*	NM_001317974.1	GGACCTCGCCTCTCTTGACAG	ACAGCAGCATTAGCAACCAACAC
*SlCAO*	XM_004241981.4	GCTATTGCTACTTCTGCTGCTCTC	CCGCTGCCTCCTCATACACTG
*SlCLH2*	XM_010328100.3	GGCGGTAACACGGCATTCG	CGTAGACAGGAGGGAGAGCATC
*SlPPH*	XM_004240993.4	TCCGCCATCAGTTCTCACCTAC	CCAAACCCGAGCCAATTACCATC
*SlPAO*	XM_004241981.4	GCTATTGCTACTTCTGCTGCTCTC	CCGCTGCCTCCTCATACACTG
*SlSGR*	EU414632.1	GGTCCACTCAGAGATGCAACTTC	TCTTCACAAGGCTTTGGTAATTCCC
*SlGGPPS*	NM_001366706.1	GTGAGTGCTCTGCCGTTTGTG	TTGTTCTCCGTCTTCTTTGCTTCC
*SlPSY1*	NM_001247883.2	TGAAGGAATGCGTATGGACTTGAG	CCAACCGTACCAGCAACATAATAAC
*SlPSY2*	NM_001247742.2	ATCCGACACTGTTTCCAGATTTCC	TTCCACAAGTCCATACGCATTCC
*SlPDS*	NM_001247166.2	TTTGTGTTTGCCGCTCCAGTG	AGGTACTCCGACTAACTTCTCCAAC
*SlLCY-B*	NM_001247297.2	GTCGTTGGAATCGGTGGTACAG	TGGCATTGGCAACAACAGGAG
*SlActin*	NM_001330119.1	ATTGTGTTGGACTCTGGTGATGGTG	GACGGAGAATGGCATGTGGAAGG

### 2.3 Data processing

Data were processed using SPSS 23.0 (IBM Co., USA) to calculate means. An independent-samples *t*-test was used for variance analysis, with the significance level set at *p* < 0.05. Graphs were generated using Origin 2022 (Origin Lab Co., USA).

## 3 Results

### 3.1 Exogenous ALA regulates tomato fruit skin color parameters

**Figure 2:**
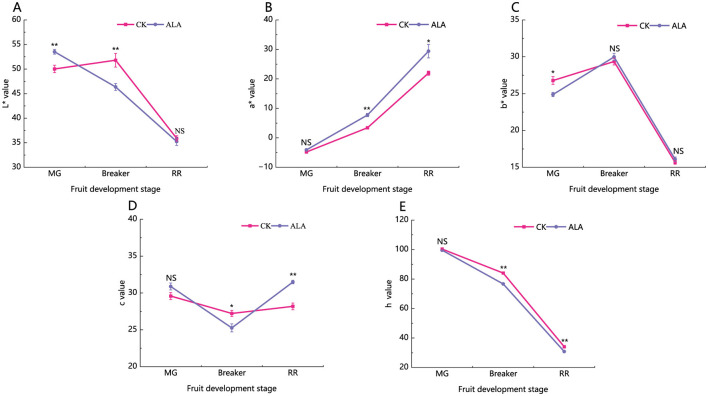
Effects of exogenous ALA on peel color parameters of tomato fruit. **(A)** L* value, **(B)** a* value, **(C)** b* value, **(D)** c value, **(E)** h value. *denote significant difference at 0.05 level, **denote significant difference at 0.01 level. NS denote that the difference is not significant.

In the control group, the L^*^ value showed a low-high-low trend during the three stages of fruit development. Exogenous ALA treatment significantly increased the L^*^ value during the mature green stage, significantly decreased it during the breaker stage, and showed no significant difference during the red ripe stage (Figure 2A). The a^*^ value gradually increased. In the mature green stage, the a^*^ value was negative, while no difference occurred at the mature green stage, ALA markedly enhanced a^*^ from the breaker stage onward, indicating accelerated red coloration. The b^*^ value remained positive. In the mature green stage, ALA slightly reduced b^*^ at the mature green stage, but no treatment effect was observed thereafter. For the c^*^ value, ALA had no impact at the mature green stage, slightly reduced it at the breaker stage, and significantly enhanced it at ripening. The h value consistently declined during maturation, and ALA further reduced h at the breaker and ripening stages (Figure 2). Overall, the increase in a^*^ and c^*^, together with the decrease in h under ALA treatment, demonstrates that exogenous ALA promotes tomato fruit coloration by modulating pigment-related changes during ripening.

### 3.2 Exogenous ALA regulates chlorophyll biosynthesis and degradation in tomato fruits

**Figure 3:**
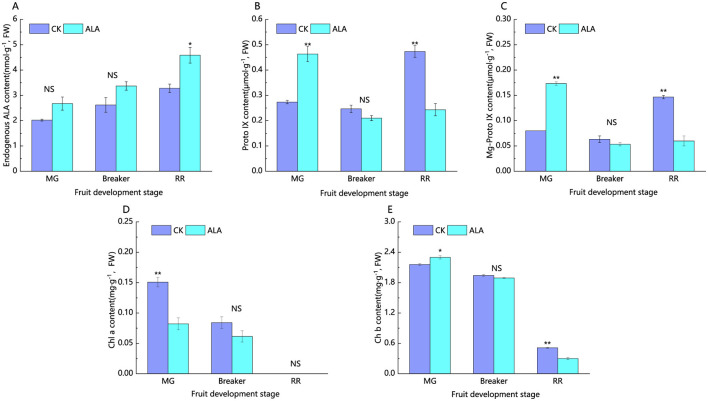
Effects of exogenous ALA on the content of intermediates in chlorophyll synthesis pathway of tomato fruit. **(A)** Endogenous ALA content, **(B)** Proto IX content, **(C)** Mg-Proto IX content, **(D)** Chl a content, and **(E)** Chl b content. *denote significant difference at 0.05 level, **denote significant difference at 0.01 level. NS denote that the difference is not significant.

Endogenous ALA in tomato fruit gradually accumulated during ripening (Figure 3A). Under exogenous ALA treatment, the endogenous ALA content increased to varying degrees. Compared to the control, endogenous ALA content was elevated by 32.3%, 28.7%, and 38.8% during the mature green, breaker, and red ripe stages, respectively.

As shown in Figures 2B, C, protoporphyrin IX and Mg-protoporphyrin IX, as intermediates in the chlorophyll biosynthesis pathway, exhibited a trend of initial decrease followed by an increase during tomato fruit ripening. During the mature green stage, 200 mg·L^−1^ALA treatment significantly increased the content of protoporphyrin IX and Mg-protoporphyrin IX. During the breaker stage, their contents decreased slightly under 200 mg·L^−1^ALA treatment, with no significant difference compared to the control. In the red ripe stage, 200 mg·L^−1^ALA treatment significantly reduced the content of protoporphyrin IX and Mg-protoporphyrin IX in the fruit (Figures 3B, C).

Chlorophyll a and b continuously declined as fruits ripened, with chlorophyll a becoming undetectable at maturity. ALA treatment accelerated chlorophyll a reduction and temporarily enhanced chlorophyll b at the mature green stage, followed by a decline during later stages (Figures 3D, E).

**Figure 4:**
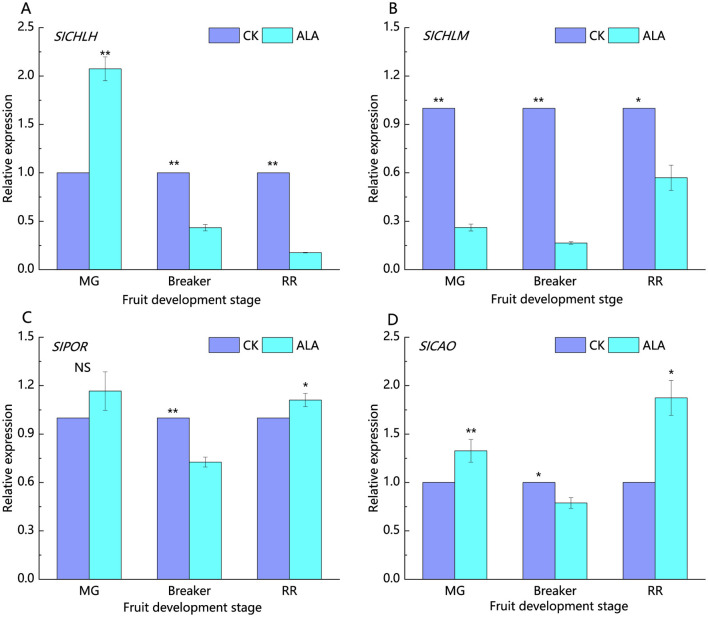
Effects of exogenous ALA on the relative expression of key genes of chlorophyll synthesis in tomato fruit. **(A)** The relative expression of *SlCHLH*, **(B)** The relative expression of *SlCHLM*, **(C)** The relative expression of *SlPOR*, **(D)** The relative expression of *SlCAO*. *denote significant difference at 0.05 level, **denote significant difference at 0.01 level. NS denote that the difference is not significant.

*SlCHLH, SlCHLM, SlPOR*, and *SlCAO* are key genes encoding enzymes involved in the chlorophyll synthesis pathway (Figure 4). During the mature green stage, treatment with 200 mg·L^−1^ ALA significantly upregulated *SlCHLH* and *SlCAO* expression. In the breaker stage, the expression levels of *SlCHLH, SlCHLM, SlPOR*, and *SlCAO* were significantly downregulated by the 200 mg·L^−1^ ALA treatment. At the red ripe stage, 200 mg·L^−1^ ALA treatment significantly upregulated *SlPOR* and *SlCAO* expression, while *SlCHLH* and *SlCHLM* were downregulated.

**Figure 5:**
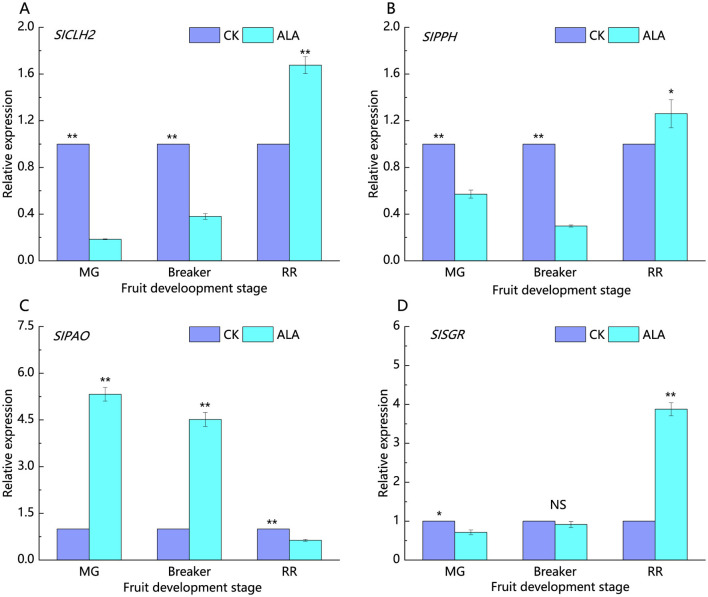
Effects of exogenous ALA on the relative expression of key genes in chlorophyll degradation pathway of tomato fruit. **(A)** The relative expression of *SlCLH2*, **(B)** The relative expression of *SlPPH*, **(C)** The relative expression of *SlPAO*, **(D)** The relative expression of *SlSGR*. *denote significant difference at 0.05 level, **denote significant difference at 0.01 level. NS denote that the difference is not significant.

*SlCLH2, SlPPH, SlPAO*, and *SlSGR* are key enzyme-encoding genes involved in the chlorophyll degradation pathway (Figure 5). During the mature green stage, treatment with 200 mg·L^−1^ALA significantly reduced the relative expression of *SlCLH2, SlPPH*, and *SlSGR*, to 0.18, 0.57, and 0.71 times that of the control, respectively. At the same time, *SlPAO* expression was significantly upregulated by ALA treatment, being 5.34 times higher than the control. In the breaker stage, the relative expression levels of *SlCLH2* and *SlPPH* were significantly lower than in the control, while *SlPAO* expression was significantly higher, at 4.52 times the control level. *SlSGR* expression showed no significant difference compared to the control in this stage. At the red ripe stage, 200 mg·L^−1^ALA treatment significantly upregulated the expression of *SlCLH2, SlPPH*, and *SlSGR*, while *SlPAO* expression was downregulated compared to the control.

### 3.3 Exogenous ALA regulates carotenoid biosynthesis and accumulation in tomato fruits

**Figure 6:**
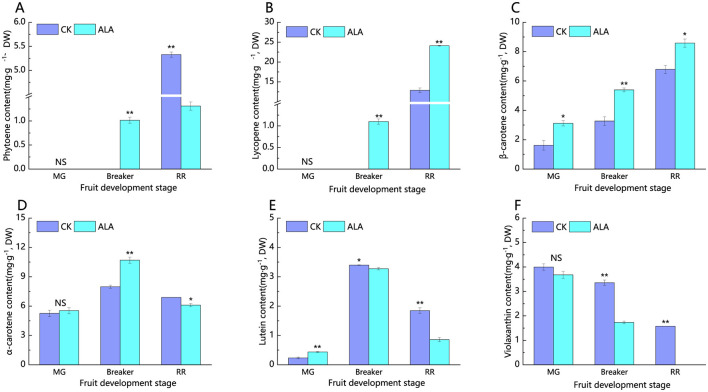
Effects of exogenous ALA on the content of carotenoid components in tomato fruit. **(A)** Phytoene content, **(B)** lycopene content, **(C)** β-carotene content, **(D)** α-carotene content, **(E)** lutein content, and **(F)** violaxanthin content. *denote significant difference at 0.05 level, **denote significant difference at 0.01 level. NS denote that the difference is not significant.

In tomato fruits, a total of six carotenoids were identified (Figure 6). As shown in Figures 6A, B, neither lycopene nor phytoene were detected in both the control and ALA-treated fruits during the green mature stage. However, at the maturity stage, exogenous ALA markedly enhanced lycopene accumulation while reducing phytoene levels.

As shown in Figure 6C, β-carotene progressively accumulates during tomato fruit maturation, and exogenous ALA treatment significantly increases the β-carotene content. Compared to the control, β-carotene content increased at both the mature green, breaker and red ripe stages. From Figure 6D, it can be seen that α-carotene content initially increases and then decreases as the tomato fruit matures. During the mature green stage, there was no significant difference in α-carotene content between the control and ALA-treated groups. However, in the breaker stage, exogenous ALA treatment significantly increased the α-carotene content, while at the red ripe stage, ALA application resulted in a reduction compared with the control.

From Figure 6E, it can be observed that lutein content in tomato fruits follows a trend of initially increasing and then decreasing during the fruit maturation process. In the mature green stage, lutein content was relatively low, but exogenous ALA treatment significantly increased its content. However, in both the breaker stage and red ripe stage, ALA treatment significantly reduced lutein levels compared to the control. As seen in Figure 6F, violaxanthin content showed a gradual decline throughout the ripening process, and by the red ripe stage, no violaxanthin was detected in the treated fruits. During the mature green stage, there was no noticeable difference in violaxanthin content between the control and ALA-treated fruits. However, in the breaker stage, ALA treatment significantly reduced violaxanthin content compared to the control.

**Figure 7:**
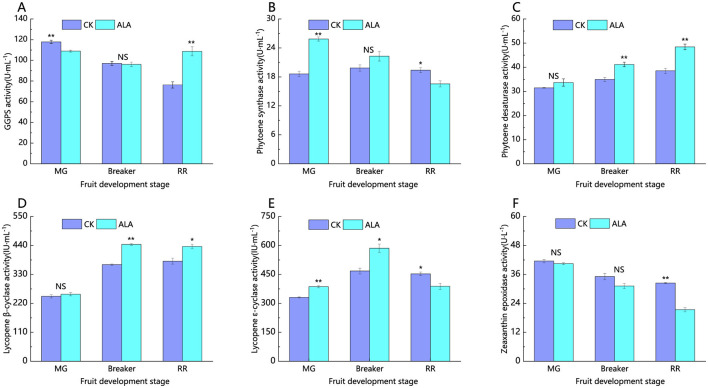
Effects of exogenous ALA on the activities of enzymes related to carotenoid synthesis in tomato fruit. **(A)** GGPS activity, **(B)** Phytoene synthase activity, **(C)** Phytoene desaturase activity, **(D)** Lycopene β-cyclase activity, **(E)** Lycopene ε-cyclase activity, and **(F)** Zeaxanthin epoxidase activity. *denote significant difference at 0.05 level, **denote significant difference at 0.01 level. NS denote that the difference is not significant.

The activity of enzymes involved in the carotenoid biosynthesis pathway plays a crucial role in regulating pigment metabolism during tomato fruit development, thereby impacting the fruit's commercial quality. As shown in Figure 7, the activities of geranylgeranyl pyrophosphate synthase (GGPS) and zeaxanthin epoxidase (ZEP) gradually decrease as the tomato fruit matures. On the other hand, the activities of phytoene desaturase (PDS), phytoene synthase (PSY), lycopene β-cyclase (LCYB), and lycopene ε-cyclase (LCYE) show an increasing trend throughout fruit development. During the green mature stage, the application of ALA significantly reduced the activity of GGPS in tomato fruit. At the same time, ALA treatment significantly increased the activities of PSY and LCYE. In the breaker stage, the activities of GGPS and ZEP under ALA treatment showed no significant difference from the control. However, the activities of PDS, PSY, LCYB, and LCYE all increased to varying degrees. In the red ripe stage, ALA treatment significantly increased the activities of GGPS, PDS, and LCYB. Conversely, ALA treatment significantly decreased the activities of PSY, LCYE, and ZEP.

**Figure 8:**
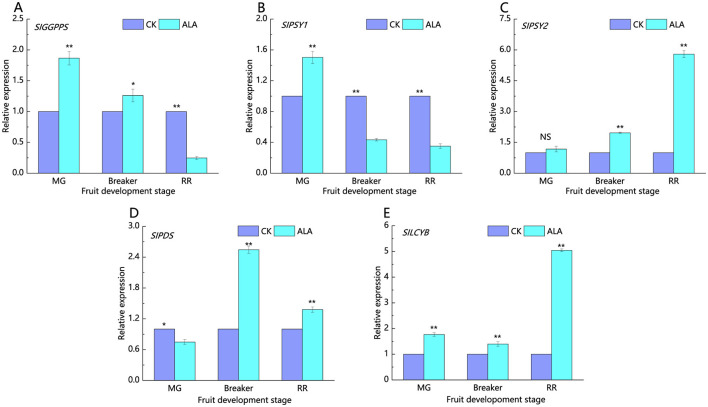
Effects of exogenous ALA on the expression of key genes involved in carotenoid synthesis in tomato fruit. **(A)** The relative expression of *SlGGPPS*, **(B)** The relative expression of *SlPSY1*, **(C)** The relative expression of *SlPSY2*, **(D)** The relative expression of *SlPDS*, **(E)** The relative expression of *SlLCYB*. *denote significant difference at 0.05 level, **denote significant difference at 0.01 level. NS denote that the difference is not significant.

*SlGGPPS, SlPSY1, SlPSY2, SlPDS*, and *SlLCYB* are key enzyme-encoding genes involved in the carotenoid biosynthesis pathway (Figure 8). At the mature green stage, treatment with 200 mg·L^−1^ALA significantly increased the relative expression levels of *SlGGPPS, SlPSY1*, and *SlLCYB* to 1.87, 1.50, and 1.77 times those of the control, respectively. In contrast, *SlPDS* expression was significantly downregulated, while *SlPSY2* expression showed no significant difference compared to the control. Breaker stage: 200 mg·L^−1^ALA treatment significantly upregulated the expression of *SlGGPPS, SlPSY2, SlPDS*, and *SlLCYB*, while *SlPSY1* expression was significantly lower than the control. Red ripe stage: 200 mg·L^−1^ALA treatment significantly upregulated the expression of *SlPSY2, SlPDS*, and *SlLCYB*, while *SlGGPPS* and *SlPSY1* expression were significantly downregulated.

### 3.4 Exogenous ALA regulates soluble sugar components in tomato fruits

**Figure 9:**
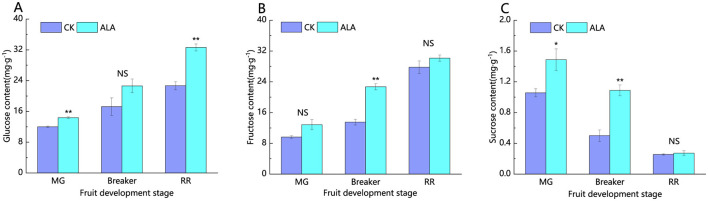
Effects of exogenous ALA on the content of soluble sugar components during tomato fruit development. **(A)** Glucose content, **(B)** fructose content, and **(C)** sucrose content. *denote significant difference at 0.05 level, **denote significant difference at 0.01 level. NS denote that the difference is not significant.

As shown in Figure 9, during tomato fruit maturation, the glucose and fructose contents exhibit an upward trend, while the sucrose content shows a downward trend. Under exogenous ALA treatment, the contents of glucose, fructose, and sucrose in tomato fruits at all stages were increased to varying degrees compared to the control.

In the mature green stage, the glucose content in both the control and treatment groups was higher than the fructose content, with sucrose being the lowest. ALA treatment significantly increased glucose and sucrose contents, with glucose and sucrose levels rising, respectively, compared to the control, while the increase in fructose content was not significant. In the breaker stage, glucose content in control fruits remained higher than fructose, and ALA treatment significantly boosted fructose and sucrose contents. Compared to the control, ALA treatment increased glucose, fructose, and sucrose contents, respectively. In the red ripe stage, ALA treatment significantly enhanced glucose content, with glucose, fructose, and sucrose levels increasing.

### 3.5 Exogenous ALA regulates organic acid components in tomato fruits

**Figure 10:**
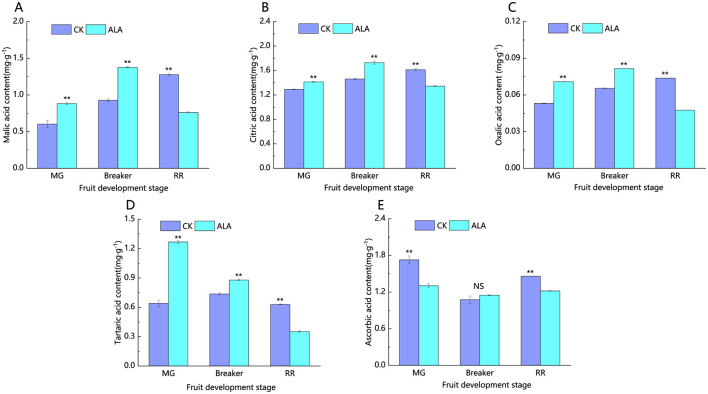
Effects of exogenous ALA on the content of organic acid components during tomato fruit development. **(A)** Malic acid content, **(B)** citric acid content, **(C)** oxalic acid content, **(D)** tartaric acid content, and **(E)** ascorbic acid content. *denote significant difference at 0.05 level, **denote significant difference at 0.01 level. NS denote that the difference is not significant.

As shown in Figure 10, the contents of malic acid, citric acid, and oxalic acid exhibited an increasing trend during the ripening of tomato fruits. In the green mature and breaker stages, exogenous ALA treatment significantly increased the levels of malic acid, citric acid, oxalic acid, and tartaric acid in the tomato fruits. During the red ripe stage, exogenous ALA treatment significantly reduced the contents of malic acid, citric acid, oxalic acid, tartaric acid, and ascorbic acid. Compared to the control, the contents of malic acid, citric acid, oxalic acid, tartaric acid, and ascorbic acid were reduced by 40.5%, 16.6%, 35.6%, 44.2%, and 16.4%, respectively.

### 3.6 Exogenous ALA affects sugar and acid quality of tomato fruits

**Figure 11:**
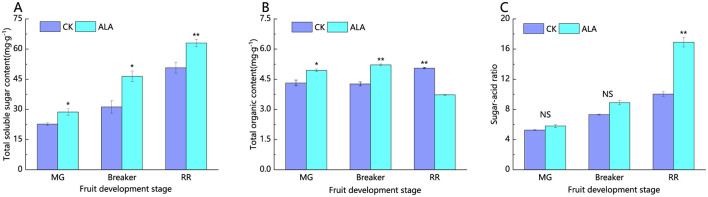
Effects of exogenous ALA on total soluble sugar, total organic acid content, and sugar-acid ratio of tomato fruit. **(A)** Total soluble sugar content, **(B)** total organic acid content, and **(C)** sugar-acid ratio. *denote significant difference at 0.05 level, **denote significant difference at 0.01 level. NS denote that the difference is not significant.

As shown in Figure 11A, exogenous ALA significantly increased the total soluble sugar content in tomato fruits at all stages. Compared to the control, the total soluble sugar content was increased by 26.5%, 48.6%, and 24.3% during the mature green stage, breaker, and red ripe stages, respectively. Under exogenous ALA treatment, the total organic acid content in tomato fruits showed an initial increase followed by a decrease (Figure 10B). The sugar-to-acid ratio displayed an upward trend. In the mature green and breaker stages, there was no significant difference in the sugar-to-acid ratio between the treatment and control groups. However, during the red ripe stage, exogenous ALA treatment significantly increased the sugar-to-acid ratio by 68.5% compared to the control (Figure 11C).

### 3.7 Correlation analysis and principal component analysis

**Figure 12:**
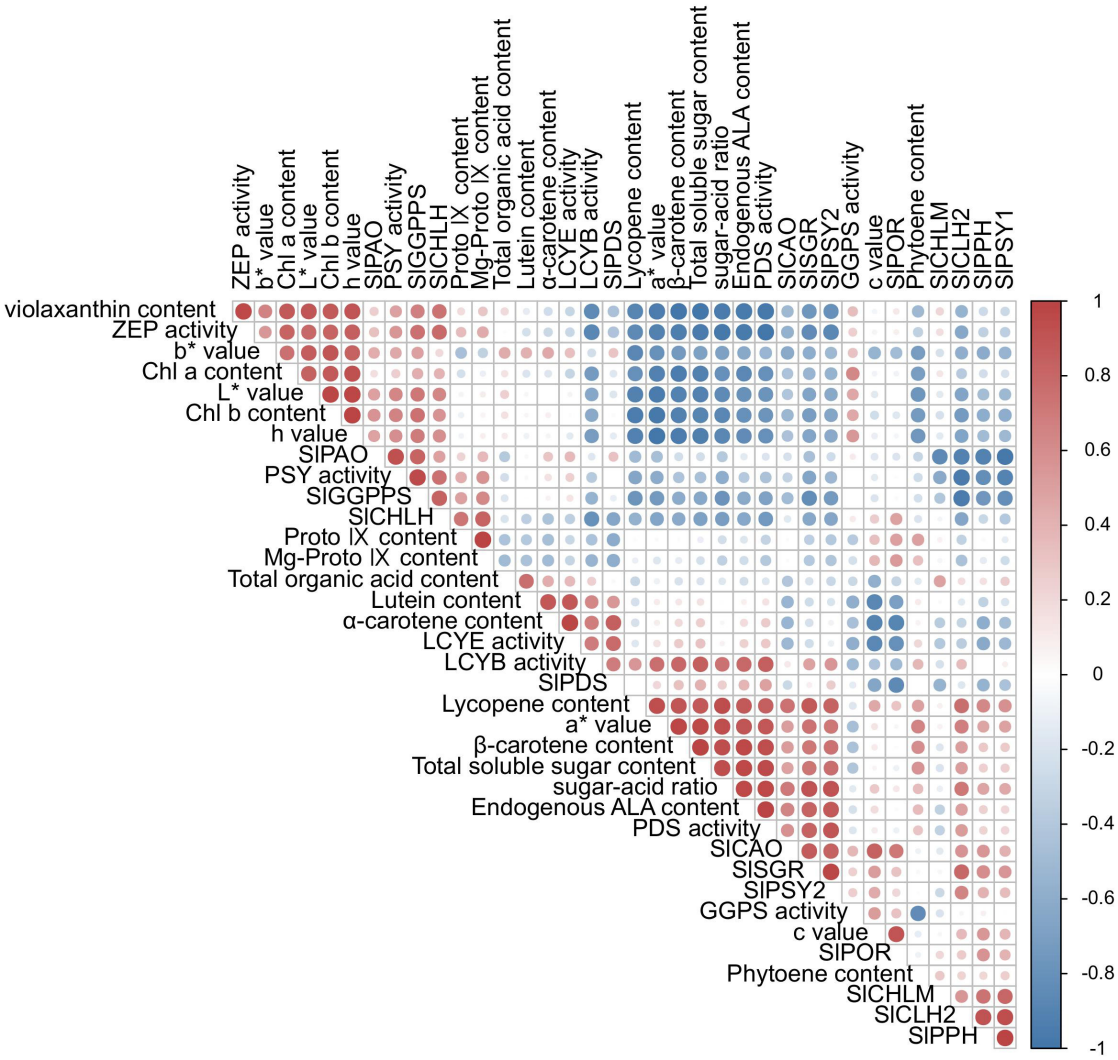
The heat map shows the correlation analysis between the observed parameters processed by ALA. Red indicates a positive correlation, blue indicates a negative correlation, and the darkness of the color represents the strength of the correlation.

Chlorophyll a and b contents, together with L^*^ and h values, showed positive correlations with chlorophyll-related genes (*SlCHLH, SlCHLM, SlPOR, SlPPH, SlCLH2*) and tetrapyrrole intermediates (Proto IX and Mg-Proto IX), while displaying negative correlations with peel color parameters (a^*^ and b^*^) (Figure 12). Key carotenoid enzymes—including PSY, PDS, lycopene β-cyclase and ε-cyclase, GGPS activity, and *SlGGPPS*—were positively correlated with downstream metabolites such as phytoene, lycopene, β-carotene, α-carotene, lutein, and violaxanthin. Lycopene content was strongly associated with a^*^ values. Notably, endogenous ALA content correlated positively with enzymes across both tetrapyrrole and carotenoid pathways, including PSY, PDS, GGPS activity, *SlCHLM*, and *SlPOR*.

## 4 Discussion

The appearance, nutritional value, and flavor quality of tomato fruits significantly affect their marketability and shelf life, playing a crucial role in product evaluation (24). The coloration of the fruit is primarily based on the content and relative proportions of pigments such as chlorophyll and carotenoids in the peel and flesh (25, 26). The content of carotenoid compounds is directly related to the nutritional value of tomatoes. Carotenoids have been shown to enhance cognitive and cardiovascular function, reduce oxidative damage, and potentially contribute to the prevention of certain cancers in human health (27, 28). Previous work by our research team applied ALA at the mature green stage and demonstrated its effectiveness in enhancing fruit coloration and improving fruit quality (13). Building on these findings, we further examined whether ALA application at the fruit setting stage could also enhance fruit yield and quality. To elucidate the underlying mechanisms, this research also analyzed the relative expression levels of key genes involved in the synthesis metabolism of chlorophyll and carotenoids, thereby providing insights into ALA induced carotenoid accumulation and chlorophyll degradation. Consistent with this, exogenous ALA in this study reduced chlorophyll content, increased lycopene and β-carotene levels, and altered color parameters (decreased L^*^ and h°, increased a^*^), indicating enhanced red coloration during ripening. In fruits with carotenoid accumulation, GGPP participates in the biosynthesis carotenoids under the action of various enzymes, thereby affecting plant coloration (29). GGPS catalyzes the conversion of FPP to GGPP, and also catalyzes the involvement of GGPP in the production of chlorophyll phytol (6). In this study, exogenous ALA treatment enhanced chlorophyll biosynthesis at the mature green stage, during which lycopene accumulation was absent. At the breaker and red ripe stages, ALA treatment promoted chlorophyll degradation, upregulated carotenoid biosynthesis-related genes, and significantly enhanced lycopene accumulation. These findings suggest that, during the early phase of tomato fruit development, cell division and fruit enlargement are the primary physiological processes, while the accumulation of lycopene is minimal or has not yet become prominent. Moreover, from the breaker stage onward, GGPS may shift its role from chlorophyll synthesis toward carotenoid biosynthesis, reflecting a progressive enhancement of the carotenoid metabolic pathway in ALA-treated fruits. These findings are similar to those observed in tomatoes and *Dunaliella* (30), where GGPS activity is closely linked to chlorophyll and carotenoid content.

The synthesis and metabolism of tomato pigments involve changes in various enzyme-related genes (31–33). *SlSGR1* encodes magnesium dechelatase and catalyzes the degradation of Chl a (34). *SlSGR1* can also interact with PSY1, a key enzyme in the carotenoid biosynthesis pathway, inhibiting PSY1 enzyme activity and thereby affecting carotenoid accumulation during tomato fruit maturation (35). It has also been suggested that the expression level of *SlLCYB* influences the accumulation of β-carotene and α-carotene in fruits (36). In this study, no phytoene was detected in either the control or treatment groups during the mature green stage. However, exogenous ALA treatment upregulated the expression of *SlSGR* during the breaker and red ripe stages of tomato fruit development, while also increasing the expression levels of *SlGGPPS* and *SlPSY2*. These results suggest that ALA may accelerate chlorophyll degradation and enhance the accumulation of lycopene and β-carotene, ultimately contributing to the significant changes in fruit coloration. By the red ripe stage, phytoene levels declined again in the ALA-treated fruits. This reduction may be related to regulatory interactions between the *SlSGR* and *SlPSY*, both of which showed elevated expression under ALA treatment. These interactions could potentially influence the flux through the carotenoid biosynthetic pathway and affect phytoene accumulation (37). Exogenous ALA influences the expression of genes related to tomato pigment biosynthesis, thereby affecting pigment synthesis. It can upregulate the expression of genes related to chlorophyll biosynthesis, such as *SlCHLM* and *SlCHLG*, thereby increasing the content of intermediate products in the chlorophyll biosynthesis pathway and promoting chlorophyll biosynthesis (38). In this study, 200 mg·L^−1^ALA treatment during the mature green stage significantly upregulated the expression of *SlCHLH* and *SlCHLM*, increasing the content of Proto IX, Mg-Proto IX, and chlorophyll b in the fruit. During the breaker and red ripe stages, exogenous ALA upregulated the expression of genes related to the chlorophyll degradation pathway (such as *SlPAO* and *SlPPH*), thereby promoting chlorophyll breakdown. It has been found that exogenous ALA significantly promoted carotenoid accumulation in tomato fruits by upregulating the expression of *SlGGPPS, SlPSY1*, and *SlLCYB* (18). In this study, 200 mg·L^−1^ALA treatment significantly increased the activities of PSY, LCYB, and PDS at various stages and upregulated the expression of carotenoid biosynthesis-related genes, such as *SlGGPPS, SlPSY*, and *SlLCYB*, thereby promoting carotenoid accumulation in the fruits (18). ALA mediates the metabolic transition from chlorophyll turnover toward carotenoid accumulation by influencing shared precursors such as GGPP and potentially coordinating *SlSGR* and *SlPSY* interactions.

Fruit ripening involves not only changes in fruit coloration but also significant alterations in fruit quality components (17). Sugars and organic acids are important carbon metabolites in plants and play a crucial role in contributing to the flavor quality of the fruit. Our previous study found that the ALA promote the soluble sugar content and reduce the organic acid content in the fruits of tomatoes cultivated in solar greenhouse (13). Fruit quality also changed markedly during ripening. The glucose, fructose, and sucrose contents were increased by ALA, meanwhile, the malic acid and citric acid contents were decreased, resulting in a higher sugar-to-acid ratio. These trends are consistent with findings in litchi (*Litchi chinensis*) (39) and peach (*Amygdalus subgenus*) (15). In tomato fruits, fructose has a high sweetness level while glucose has a lower sweetness level (21). Researchers have proposed that fructose and citric acid are more important than glucose and malic acid, and that the formation of optimal flavor quality in tomato fruits requires a high sugar content and relatively high acidity (40). In this study, glucose and fructose contents gradually accumulated during tomato fruit maturation. Exogenous ALA treatment significantly increased the levels of glucose, fructose, and sucrose, with glucose content being higher than fructose content in red ripe stage tomato fruits. Tomatoes are classified as citric acid-accumulating fruits (41). During tomato fruit maturation, relatively high levels of citric acid were consistently observed. In this study, the application of 200 mg·L^−1^exogenous ALA significantly reduced the contents of citric acid, malic acid, tartaric acid, oxalic acid, and ascorbic acid in red ripe stage tomato fruits.

A description of the limitations of this study is provided. To ensure data reliability, the experiments were conducted under controlled conditions using a single tomato cultivar and a fixed concentration of ALA, which may limit the generalizability of the results to other genotypes or cultivation environments. Moreover, while transcript levels and enzyme activities were measured, direct evidence linking these molecular changes to metabolite accumulation remains correlative. Future research incorporating metabolomics, proteomic profiling, and transgenic validation of key genes will help clarify the mechanistic basis of ALA-mediated pigment regulation. Such approaches are also necessary to determine whether ALA influences plastid development or hormone cross-talk.

## 5 Conclusion

**Figure 13:**
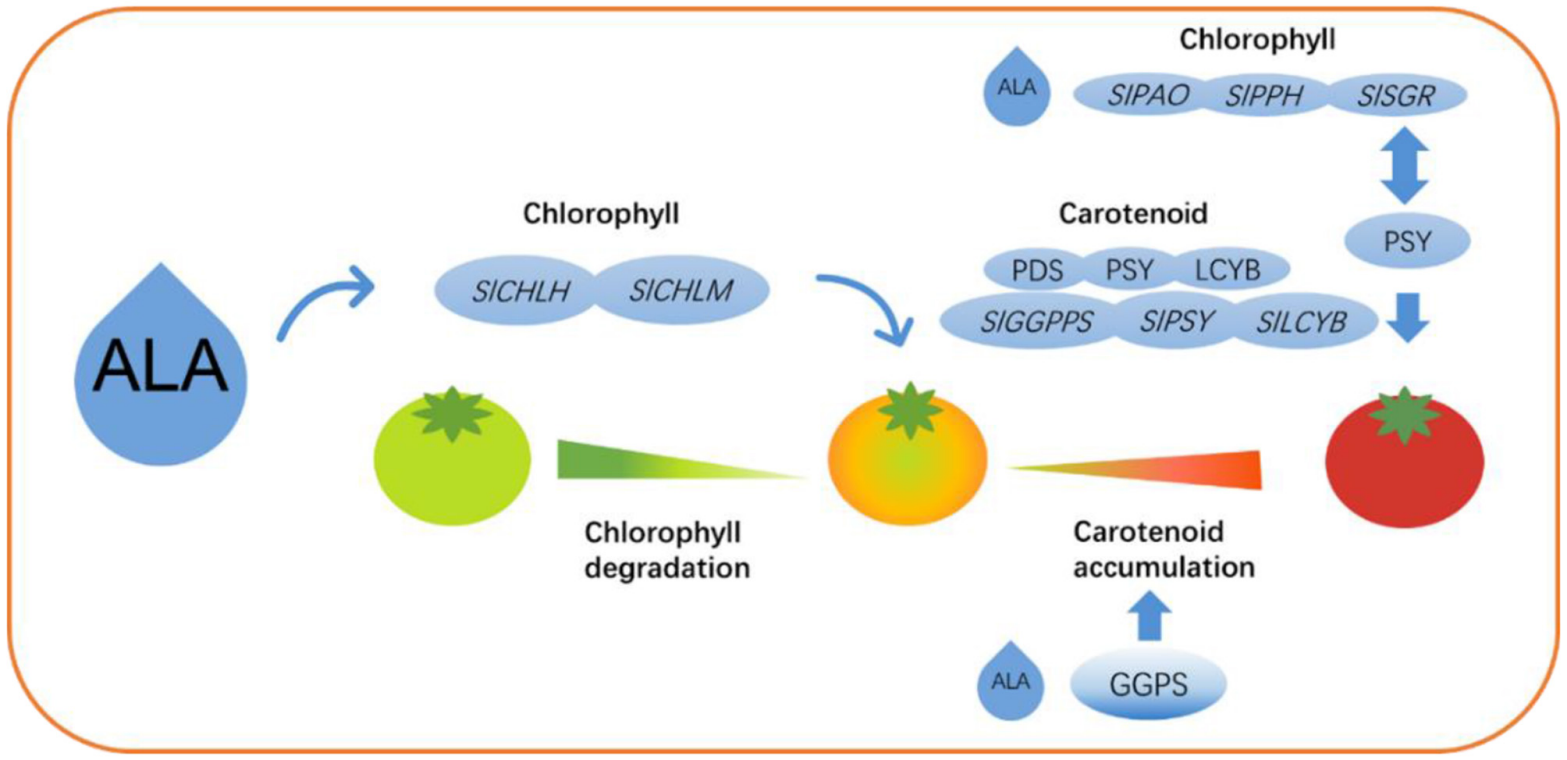
A proposed working model of ALA in regulating carotenoid accumulation and fruit ripening. At the mature-green stage, exogenous ALA enhanced chlorophyll accumulation by upregulating chlorophyll biosynthesis genes (*SlCHLH, SlCHLM*), increasing biosynthetic intermediates, and reducing GGPS activity to suppress chlorophyll degradation. In contrast, at the breaker and red-ripe stages, ALA promoted chlorophyll breakdown by upregulating degradation-related genes (*SlPAO, SlPPH, SlSGR*), while increasing GGPS activity to shift metabolic flux toward carotenoid biosynthesis. During these stages, ALA also markedly enhanced the activities and expression levels of key carotenoid biosynthetic enzymes and genes (PDS, PSY, LCYB; *SlGGPPS, SlPSY, SlLCYB*).

The present study has demonstrated that treatment with 200 mg·L^−1^ALA accelerate chlorophyll degradation during tomato fruit maturation by upregulating chlorophyll degradation-related genes (such as *SlCLH2, SlPPH*, and *SlSGR*) and reducing the content of intermediate products involved in chlorophyll biosynthesis. ALA also enhances the activity of enzymes related to carotenoid biosynthesis (such as PDS, PSY, and LCYB) and upregulates the expression of related genes (such as *SlGGPPS, SlPSY*, and *SlLCYB*), thereby promoting carotenoid accumulation and influencing the color changes in tomato fruits (Figure 13). Additionally, 200 mg·L^−1^ALA significantly increased the glucose and fructose content while reducing malic and citric acid levels, resulting in an increased sugar-to-acid ratio that could potentially enhance flavor quality.

## Data Availability

The original contributions presented in the study are included in the article/supplementary material, further inquiries can be directed to the corresponding author/s.
